# Minimum Inhibitory Concentrations before and after Antibacterial Treatment in Patients with Mycobacterium abscessus Pulmonary Disease

**DOI:** 10.1128/Spectrum.01928-21

**Published:** 2021-12-08

**Authors:** Keiji Fujiwara, Fumiko Uesugi, Koji Furuuchi, Yoshiaki Tanaka, Takashi Yoshiyama, Mikio Saotome, Ken Ohta, Satoshi Mitarai, Kozo Morimoto

**Affiliations:** a Respiratory Disease Center, Fukujuji Hospital, Japan Anti-Tuberculosis Association, Tokyo, Japan; b Department of Mycobacterium Reference and Research, The Research Institute of Tuberculosis, Japan Anti-Tuberculosis Association, Tokyo, Japan; c Department of Basic Mycobacteriosis, Nagasaki University Graduate School of Biomedical Sciences, Nagasaki, Japan; d Division of Clinical Research, Fukujuji Hospital, Japan Anti-Tuberculosis Association, Tokyo, Japan; Johns Hopkins University School of Medicine

**Keywords:** *Mycobacterium abscessus* species, *Mycobacterium abscessus* subsp. *abscessus*, *Mycobacterium abscessus* subsp. *massiliense*, rapidly growing mycobacteria, drug susceptibility test, NTM, nontuberculous mycobacteria

## Abstract

The clinical importance of Mycobacterium abscessus (MABS) pulmonary disease has been increasing. However, there is still a lack of information about MIC distribution patterns and changes in clinical practice settings. The MIC results of rapidly growing mycobacteria isolated from 92 patients with nontuberculous mycobacterial pulmonary disease diagnosed from May 2019 to March 2021 were retrospectively analyzed. Most of the patients (86 patients; 93.5%) were infected with MABS; 46 with Mycobacterium abscessus subsp. *abscessus* (Mab), and 40 with Mycobacterium abscessus subsp. *massiliense* (Mma). Significant differences in susceptibility to clarithromycin (15.2% versus 80.0%, *P < *0.001) and azithromycin (8.7% versus 62.5%, *P < *0.001) were observed between Mab and Mma. Most isolates were susceptible to amikacin (80; 93.0%), and over half were susceptible to linezolid (48; 55.8%). Only one-quarter of isolates (22, 25.6%) were susceptible to imipenem, while more than half (56; 65.1%) had intermediate susceptibility. Fifty-one isolates (59.3%) had MIC values of less than 1 μg/mL for sitafloxacin, which were significantly higher than isolates for moxifloxacin (5; 5.8%), especially in Mab. Sixty-five (75.6%) isolates had MICs of less than 0.5 μg/mL to clofazimine. Two patients showed obvious MIC result changes: from susceptible to resistant to clarithromycin and from resistant to susceptible to amikacin and imipenem. In conclusion, MABS isolates were relatively susceptible to amikacin and linezolid, and clarithromycin and azithromycin were especially effective against Mma. In addition, sitafloxacin and clofazimine had low MICs and might be effective treatment agents.

**IMPORTANCE** The MICs of isolates from 86 patients with Mycobacterium abscessus (MABS); 46 with Mycobacterium abscessus subsp. *abscessus* (Mab), and 40 with Mycobacterium abscessus subsp. *massiliense* (Mma) were retrospectively analyzed. The main findings are as follows: (i) Mma were significantly more susceptible to clarithromycin and azithromycin than Mab, and both subspecies tended to be more susceptible to clarithromycin than azithromycin. (ii) Most isolates were susceptible to amikacin (93.0%), and over half to linezolid (55.8%). (iii) Fifty-one isolates (59.3%) had MIC values of less than 1 μg/mL for sitafloxacin, and 65 (75.6%) had less than 0.5 μg/mL for clofazimine, which seems worth clinical investigating. (iv) Among nine cases analyzed chronological changes, only two patients showed obvious MIC result changes even after the long-term multidrug treatment. The present study revealed MICs of MABS clinical isolates before and after treatment in clinical settings, which could help develop future MABS treatments strategies.

## INTRODUCTION

The prevalence of nontuberculous mycobacterial (NTM) infections has increased worldwide and has been recognized as an important public health issue (1). Mycobacterium abscessus (MABS), a rapidly growing mycobacteria (RGM), is the second most prevalent mycobacterium in Asia, and its prevalence is increasing in Japan (1, 2). High rates of inducible resistance due to the inducible methyltransferase coded by the *erm*(41) gene are associated with low treatment success rates of 45–53% (3–5). However, it has been shown that the treatment success rates are higher in Mycobacterium abscessus subsp. *massiliense* (Mma) than in Mycobacterium abscessus subsp. *abscessus* (Mab) because the former generally has a truncated and therefore nonfunctional *erm*(41) gene (5, 6). In addition, the T28C sequevar confers susceptibility in both subspecies.

The current guidelines recommend an initial macrolide-containing, three-drug regimen to treat infection caused by isolates susceptible to mutational and inducible resistance mechanisms (Mma and T28C sequavar without *rrl* mutation) (7). However, if the isolates contain the intact *erm*(41) gene or *rrl* mutation, more than four nonmacrolide drugs are suggested during the intensive treatment phase (7). Amikacin (AMK) is a central component of the regimens, and it is recommended in both the initial (infusion) and continuation (inhalation) phases (7). The *rrs* mutation causes AMK resistance and is associated with treatment failure (8). Based on this evidence, macrolide and AMK susceptibility testing are recommended by the guidelines (7).

Imipenem (IPM) usually has a relatively low MIC and has been recommended since the issuance of the former American Thoracic Society/Infectious Diseases Society of America statement (9). Although no comparative study or clinical case series has been reported, a recent systematic review showed the efficacy of IPM-containing regimens (3). MABS often show high MIC values against many antibiotics; however, MICs vary among isolates, suggesting that MIC tests may be essential in drug selection. The guidelines recommend several drugs, including clofazimine (CLO) and linezolid (LZD), in the regimens, possibly based on MIC value distributions and scarce clinical data (7). Furthermore, the Clinical and Laboratory Standards Institute (CLSI) recommends that a drug susceptibility test (DST) should be performed using a broth microdilution-based panel (10). The MIC data of clinical isolates, which are different by isolates, should be analyzed for future regimen development.

Most previously reported MIC data from Japan were obtained from stored isolates at specialized medical institutions for NTM research, their clinical importance was unclear (11, 12). Since 2019, a commercially available MIC kit (Broth MIC RGM; Kyokuto Pharmaceutical Industry Co., Ltd., Tokyo, Japan), compliant with the CLSI M24 3rd ed recommendation, has been implemented in clinical settings in Japan. Accordingly, this study aimed to investigate the MIC distributions of MABS in a tertiary hospital setting. We also studied the MIC changes before and after long-term multidrug treatment.

## RESULTS

### Characteristics of the study patients.

Of the 92 RGM patients, 46 were infected with Mab, 40 with Mma, three with Mycobacterium fortuitum, and one each with Mycobacterium chelonae, Mycobacterium mageritense, and Mycobacterium mucogenicum. Table 1 shows the characteristics of the MABS patients. Seventy-four patients (86.0%) were female, and 62 (72.1%) had never smoked. When comparing Mab and Mma, Mab patients were older (69 versus 62 years old, *P = *0.039) and had a higher rate of previous NTM pulmonary disease history (65.2 versus 37.5%, *P = *0.017) than Mma patients. The characteristics of the other RGM patients are shown in Table S1 in the supplemental material.

**TABLE 1:** Baseline characteristics of MABS patients^*a*^

Characteristic	MABS (*n* = 86)	Mab (*n* = 46)	Mma (*n* = 40)	*P* value
Female	74 (86.0)	38 (82.6)	36 (90.0)	0.367
Age	66 (57–73)	69 (59–77)	62 (57–69)	0.039
Body mass index, kg/m^2^	19.2 (17.2–21.4)	19.6 (17.2–21.6)	18.8 (17.2–20.2)	0.359
Smoking history				
Never smoker	62 (72.1)	33 (71.7)	29 (72.5)	1
Respiratory disease				
Previous tuberculosis	6 (7.0)	3 (6.5)	3 (7.5)	1
Previous NTM pulmonary disease	45 (52.3)	30 (65.2)	15 (37.5)	0.017
Aspergillus	7 (8.1)	3 (6.5)	4 (10.0)	0.700
Systemic disease				
Diabetes mellitus	3 (3.5)	2 (4.3)	1 (2.5)	1
Gastro-intestinal disease	7 (8.1)	2 (4.3)	5 (12.5)	0.243
Radiographic findings				
Classification				
Noncavitary NB	44 (51.2)	22 (47.8)	22 (55.0)	0.782
Cavitary NB	32 (37.2)	19 (41.3)	13 (32.5)	
Fibrocavitary	7 (8.1)	3 (6.5)	4 (10.0)	
Unclassified	3 (3.5)	2 (4.3)	1 (2.5)	
Positive AFB smear	68 (97.1)	35 (76.1)	33 (82.5)	0.597

a Data are presented as *n* (%) or median (interquartile). MABS, Mycobacterium abscessus; Mab, Mycobacterium abscessus subsp. *abscessus*; Mma, Mycobacterium *abcessus* subsp. *massiliense*; NTM, nontuberculous mycobacteria; NB, Nodular bronchiectatic; AFB, Acid-fast bacilli.

### MIC profiles of MABS.

**FIG 1:**
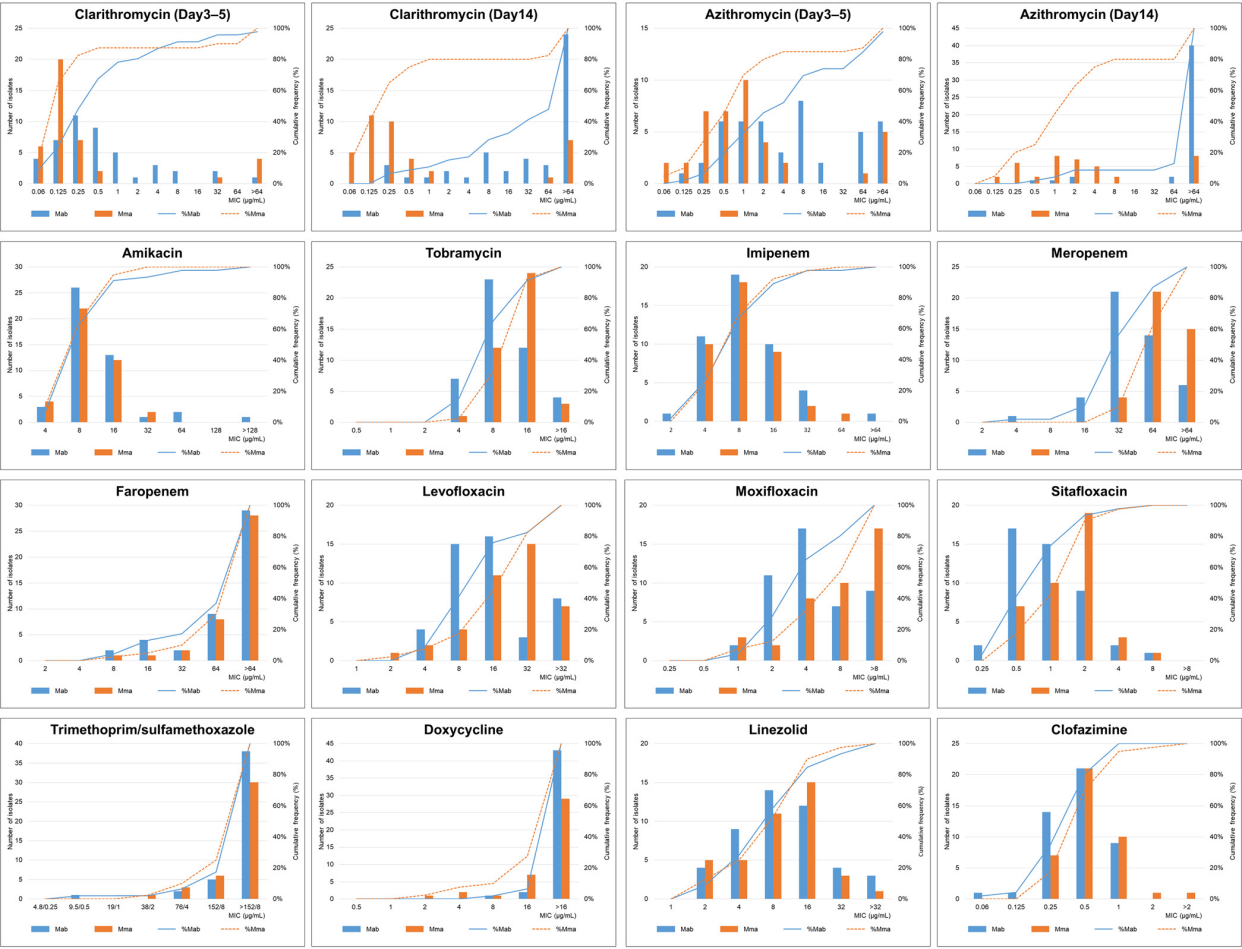
Distribution of MIC values for each drug tested in this study and cumulative percentages of inhibited isolates in 86 clinical isolates of Mab and Mma. MIC values for clarithromycin and azithromycin (days 3–5) were not recorded in one Mab isolate (Clarithromycin MIC for day 14 was 0.25). Mab, Mycobacterium abscessus subsp. *abscessus*; Mma, Mycobacterium abscessus subsp. *massiliense*.

Less than half of the isolates were susceptible to clarithromycin (CLR) (39 isolates, 45.3%) and azithromycin (AZM) (29 isolates, 33.7%) (Table 2). Thirty-six (41.9%) MABS isolates were inducible resistant to CLR, and 10 (11.6%) isolates were acquired resistant. Most isolates were susceptible to AMK (80 isolates, 93.0%), and over half were susceptible to LZD (48 isolates, 55.8%). Only one-quarter of isolates (22 isolates, 25.6%) were susceptible, and 56 isolates (65.1%) had intermediate susceptibility to IPM. In addition, only five isolates (5.8%) were susceptible to moxifloxacin (MXF) with MIC values less than 1 μg/mL, whereas 51 (59.3%) isolates had MIC values less than 1 μg/mL for sitafloxacin (STX) (*P < *0.001) (Fig. 1). Twenty-three (26.7%) isolates had CLO MIC values less than 0.25 μg/mL, and 65 (75.6%) isolates had CLO MIC values less than 0.5 μg/mL. More than 90% of the isolates were resistant to tobramycin (TOB), meropenem (MEM), levofloxacin (LVX), trimethoprim-sulfamethoxazole (SXT), and doxycycline (DOX) (Table 2). Seventy-eight (90.7%) isolates had faropenem (FRM) MIC values higher than 32 μg/mL (Fig. 1).

**TABLE 2:** Drug susceptibility test results of MABS patients^*a*^

	MABS (86)			Mab (46)			Mma (40)		
Antibiotic	Susceptible	Intermediate	Resistant	Susceptible	Intermediate	Resistant	Susceptible	Intermediate	Resistant
Clarithromycin	39 (45.3)	1 (1.2)	46 (53.5)	7 (15.2)	1 (2.2)	38 (82.6)	32 (80.0)	0 (0.0)	8 (20.0)
Azithromycin	29 (33.7)	5 (5.8)	52 (60.5)	4 (8.7)	0 (0.0)	42 (91.3)	25 (62.5)	5 (12.5)	10 (25.0)
Amikacin	80 (93.0)	3 (3.5)	3 (3.5)	42 (91.3)	1 (2.2)	3 (6.5)	38 (95.0)	2 (5.0)	0 (0.0)
Tobramycin	0 (0.0)	8 (9.3)	78 (90.7)	0 (0.0)	7 (15.2)	39 (84.8)	0 (0.0)	1 (2.5)	39 (97.5)
Imipenem	22 (25.6)	56 (65.1)	8 (9.3)	12 (26.1)	29 (63.0)	5 (10.9)	10 (25.0)	27 (67.5)	3 (7.5)
Meropenem	1 (1.2)	4 (4.7)	81 (94.2)	1 (2.2)	4 (8.7)	41 (89.1)	0 (0.0)	0 (0.0)	40 (100)
Faropenem	N/A	N/A	N/A	N/A	N/A	N/A	N/A	N/A	N/A
Levofloxacin	0 (0.0)	1 (1.2)	85 (98.8)	0 (0.0)	0 (0.0)	46 (100)	0 (0.0)	1 (2.5)	39 (97.5)
Moxifloxacin	5 (5.8)	13 (15.1)	68 (79.1)	2 (4.3)	11 (23.9)	33 (71.7)	3 (7.5)	2 (5.0)	35 (87.5)
Sitafloxacin	N/A	N/A	N/A	N/A	N/A	N/A	N/A	N/A	N/A
Trimethoprim/sulfamethoxazole	2 (2.3)	N/A	84 (97.7)	1 (2.2)	N/A	45 (97.8)	1 (2.5)	N/A	39 (97.5)
Doxycycline	0 (0.0)	3 (3.5)	83 (96.5)	0 (0.0)	0 (0.0)	46 (100)	0 (0.0)	3 (7.5)	37 (92.5)
Linezolid	48 (55.8)	27 (31.4)	11 (12.8)	27 (58.7)	12 (26.1)	7 (15.2)	21 (52.5)	15 (37.5)	4 (10.0)
Clofazimine	N/A	N/A	N/A	N/A	N/A	N/A	N/A	N/A	N/A

a Data are presented as *n* (%). MABS, Mycobacterium abscessus; Mab, Mycobacterium abscessus subsp. abscessus; Mma, Mycobacterium abcessus subsp. massiliense; N/A, not applicable.

### Differential MIC profiles of Mab and Mma.

Seven Mab isolates (15.2%) were susceptible to CLR, whereas 32 Mma isolates (80.0%) were susceptible to CLR, with a statistically significant difference (*P < *0.001) (Table 2). A similar result was observed for AZM (8.7% versus 62.5%, *P < *0.001). Although the difference was not significant, the proportion of AZM-susceptible isolates was lower than that of CLR-susceptible isolates in both Mab (8.7% versus 15.2%, *P = *0.552) and Mma (62.5% versus 80.0%, *P = *0.137). Thirty-three (86.8%) Mab isolates were inducible resistant to CLR and five (13.2%) isolates were acquired resistant. No significant differences between Mab and Mma in susceptibility to AMK (91.3 versus 95.0%, *P = *0.681) and IPM (26.1 versus 25.0%, *P = *1.000) were observed. The STX MIC values for Mab tended to be lower than those for Mma (MIC_50/90_ 1/2 versus 2/2 μg/mL) (Fig. 1).

### Temporal changes of MIC profiles in patients with antimycobacterial treatment.

**FIG 2:**
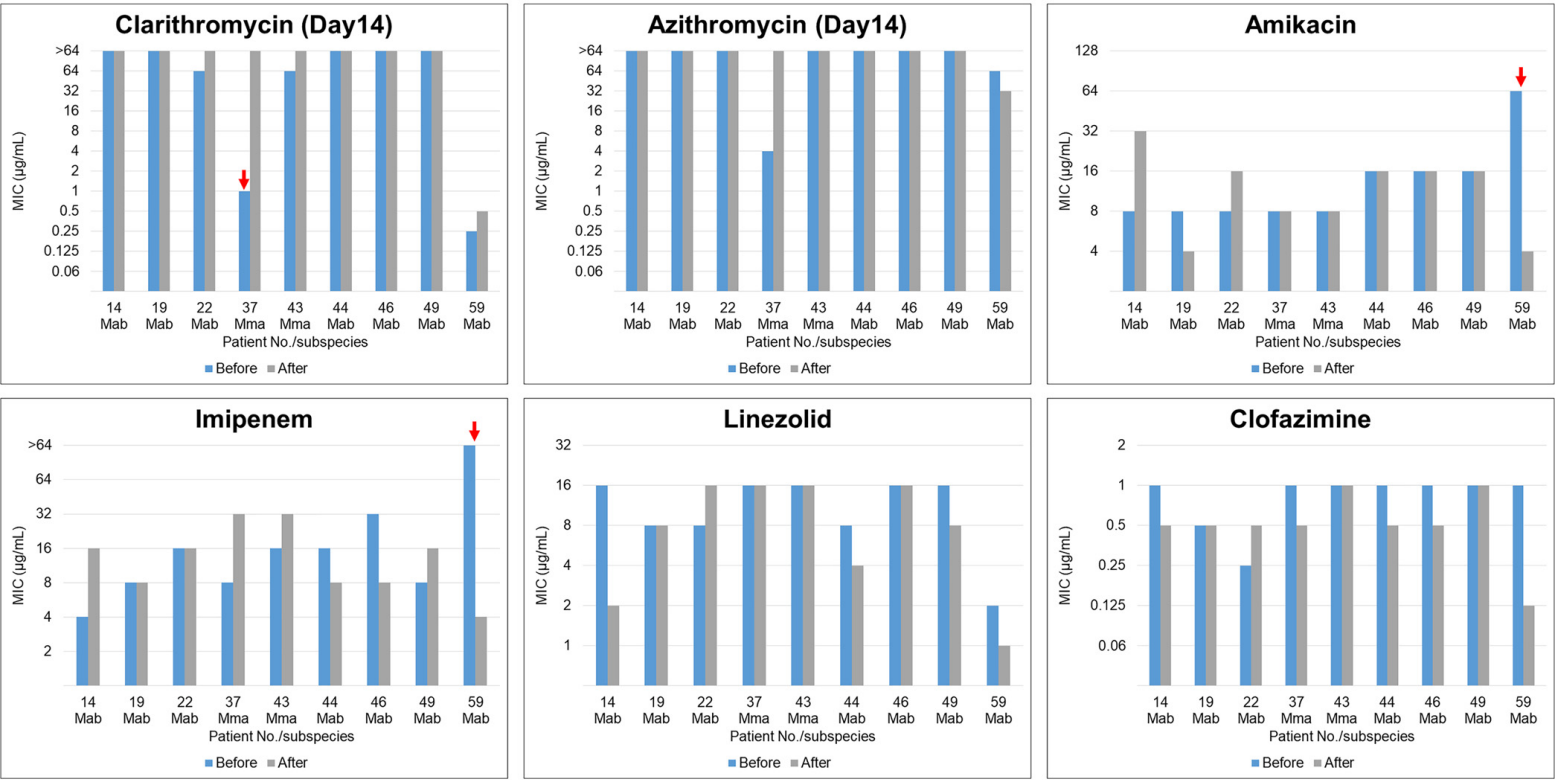
Changes in MICs of antibiotics among isolates were obtained from nine identical patients. Obvious MIC changes were observed in isolates from two cases (indicated by arrows): from susceptible to resistance to clarithromycin and from resistant to susceptible to amikacin and imipenem. Mab, Mycobacterium abscessus subsp. *abscessus*; Mma, Mycobacterium abscessus subsp. *massiliense*.

The chronological MIC changes of isolates from nine MABS patients (seven patients with Mab and two patients with Mma infection) are shown in Fig. 2. The median interval between the collections of each isolate was 284 days (range, 244–315). All nine patients were treated for a median duration of 284 days (rang, 244–315), but culture conversion (culture conversion: more than three consecutive negative cultures collected at least 4 weeks apart) was not achieved (13). The most frequently administered drugs were STX (eight patients, 88.9%), followed by macrolides (CLR and/or AZM) (seven patients, 77.8%), AMK (seven patients, 77.8%), IPM (seven patients, 77.8%), and CLO (five patients, 55.6%). The MIC results generally showed minor changes within the same susceptibility profile, susceptible–intermediate, and intermediate–resistant ranges. However, isolates from two patients showed obvious changes in MIC values, as indicated by arrows, from susceptible to resistant to CLR and from resistant to susceptible to AMK and IPM.

## DISCUSSION

In the present study, we aimed to clarify the MIC profiles in patients with RGM pulmonary disease, especially MABS, in a clinical setting. Regarding MABS, the two subspecies were observed in almost equal proportions (Mab versus Mma 53.5 versus 46.5%), with significant differences in susceptibility to macrolides. Most isolates were susceptible to AMK; however, less than 30% and 60% showed susceptibility to IPM and LZD, respectively, in both subspecies. More than 70% of isolates had CLO MIC values less than 0.5 μg/mL. Furthermore, only 5.8% of isolates were susceptible to MXF with MIC values less than 1 μg/mL, while approximately 60% of isolates had STX MIC values less than 1 μg/mL. Interestingly, regardless of persistent culture positivity under long-term multidrug treatment, most MIC data showed minor changes in susceptibility patterns. One patient changed from susceptible to resistant to CLR, and one patient changed from resistant to susceptible to IPM and AMK.

Consistent with current knowledge, Mma was more susceptible to CLR (80.0 versus 15.2%, *P < *0.001) and AZM (62.5 versus 8.7%, *P < *0.001) than Mab (14). Although the proportion of AZM-susceptible isolates tended to be lower than that of CLR-susceptible isolates, it is unknown whether there are any actual clinical differences or whether this result is due to the accuracy of the data because the CLSI has not indicated an AZM breakpoint (10). New guidelines recommend the use of AZM rather than CLR; thus, macrolide selection may need to be further investigated to strengthen the evidence (7). Among the aminoglycosides, AMK was associated with the highest susceptibility (93.0%). A systematic review showed that AMK was associated with the Mab treatment success rate (3); thus, AMK must play a central role in treating MABS pulmonary disease, especially disease caused by macrolide-resistant Mab.

Although only a quarter of isolates (22 isolates, 25.6%) were susceptible to IPM, synergistic effects of IPM with CLR and LZD were reported (15, 16), and a systematic review showed that IPM was associated with a higher success rate (3). Therefore, IPM might be justifiable for MABS pulmonary disease treatment despite the relatively low susceptibility rate. Although LZD is recommended by the guidelines (7, 17) and more than 50% of isolates were susceptible in the present study, few clinical efficacy data have been published (18), and adverse events such as myelosuppression and peripheral neuropathy are reportedly common (19, 20). Thus, we consider that analyzing pharmacokinetic data of LZD in clinical practice is warranted.

Only five (5.8%) isolates in the present study were susceptible to MXF, with MIC values less than 1 μg/mL, suggesting that MXF might not be effective in the treatment of MABS pulmonary disease (17). Significantly more isolates had MIC values less than 1 μg/mL for STX than for MXF (59.3 versus 5.8%, *P < *0.001). STX is known to have high antimycobacterial activity against Mycobacterium tuberculosis and Mycobacterium avium complex (MAC) *in vitro* (21, 22). In addition, the potential efficacy of STX for refractory MAC pulmonary disease has been reported (23). Interestingly, the MIC value of STX for Mab tended to be lower than that for Mma (MIC_50/90_ 1/2 versus 2/2 μg/mL). Although the antimycobacterial activity of STX against Mab has not been investigated *in vivo*, it might be worth investigating whether STX could be an option for the treatment of Mab pulmonary disease.

A previous study reported that CLO-containing regimens significantly decreased the rate of positivity of sputum acid-fast bacilli (AFB) culture in patients with Mab pulmonary disease (24), and isolates with CLO MIC values less than 0.25 μg/mL showed a higher rate of sputum culture conversion (25). In the present study, 23 MABS isolates (26.7%) had MIC values of less than 0.25 μg/mL. Considering the synergistic effects of CLO combined with AMK and CLR, CLO might be a practical option in the initial phase (26). Given the frequent side effects of skin and gastrointestinal symptoms and the fact that more than 30% of patients need a dose reduction, further understanding of its clinical use, including pharmacokinetics and pharmacodynamics, is needed, as lean women are the most affected, especially in Asian countries (27).

Because most of the isolates in the present study were resistant to TOB (90.7%), LVX (98.8%), SXT (97.7%), and DOX (96.5%), their efficacy in treating MABS pulmonary disease might be limited. FRM, a penem antimicrobial agent, has been used in Japan as an adjuvant in MABS treatment (28). In the present study, 90.7% of the patients had FRM MIC values of more than 32, suggesting that its antimycobacterial activity might not be promising. However, a synergistic effect with IPM *in vitro* was reported, and further *in vivo* studies are warranted (29).

MIC profile changes in nine patients with persistent culture positivity were analyzed. Unexpectedly, most of the MIC results showed minor changes. Mma became resistant to CLR, with MIC values varying from 1 to >64, in only one case of pulmonary disease. Surprisingly, the DST profile of AMK and IPM changed from resistant to susceptible in one patient. Jhun and colleagues reported that 73% of MAC pulmonary disease cases with persistent positive cultures were caused by reinfection (30). In addition, since IPM had limited stability *in vitro* and MIC values rose significantly with incubation time, different measurement day might affect the results of these susceptibility changes (31). Although we considered these strains to be single genotypes because of persistent culture positivity and no significant changes in other MICs, further large-sample studies are needed, along with basic analyses of genotypes and drug-resistant mechanisms.

Our study has several limitations. First, because the present study was conducted at a single facility using a retrospective design, the number of MIC results was small, and selection bias might have occurred in the characteristics of the patients visiting our hospital and treatment practices. Second, the DST panel used in the present study did not include cefoxitin, ciprofloxacin, or tigecycline, which are recommended to be tested by the CLSI (10). Instead, FRM, STX, and CLO were included. Third, the isolates tested for drug susceptibility twice were not shown to be genetically identical.

In conclusion, the MIC data analyses for MABS clinical isolates revealed that the guideline-recommended drugs had various MIC ranges. CLO and STX had lower MIC values, suggesting that further investigation of their efficacy in the clinical setting is needed. Because of the lack of treatment options, DST analyses for newer drugs are warranted.

## MATERIALS AND METHODS

### Collection of RGM isolates and study patients.

This retrospective study was conducted at Fukujuji Hospital, Japan Anti-Tuberculosis Association, a 340-bed facility located at the northwest end of Tokyo, Japan. A total of 112 RGM isolates underwent DST from May 2019 to March 2021. Of the 112 tests, 17 were conducted in copy isolates. Three patients who did not fulfill the criteria for the diagnosis of NTM pulmonary disease were excluded from the primary analyses (7). Accordingly, a total of 92 patients were enrolled in this study. For the patients with multiple MIC tests, primary MICs were used to construct the base cohort which was analyzed. Furthermore, we analyzed the changes in MIC after long-term treatment, focusing on frequently administered drugs (CLR, AZM, AMK, IPM, LZD, and CLO).

The Fukujuji Hospital Institutional Review Board approved the study protocol (protocol number: 20044), and informed consent was waived because of the retrospective nature of the analysis. We analyzed the medical records in April 2021.

### Microbiological examination and MIC.

Sputum AFB smears and mycobacterial culture were conducted according to standard methods (32). Isolates were identified by using matrix-assisted laser desorption/ionization time-of-flight mass spectrometry (MALDI TOF-MS) (33). MABS subspecies were identified using multiplex PCR according to a previously reported method (34).

MIC was measured according to the manufacturer’s instructions. The following 14 drugs were tested: CLR, AZM, AMK, TOB, IPM, MEM, FRM, LVX, MXF, STX, SXT, DOX, LZD, and CLO. The susceptibility results were evaluated considering the resistance cutoff points in CLSI M62 and values in the literature (Table 3) (10, 35). The MIC changes for CLR, AZM, AMK, IPM, LZD, and CLO were analyzed in nine patients in which MICs were measured twice with an interval of more than 6 months and culture conversion (culture conversion: more than three consecutive negative cultures collected at least 4 weeks apart) was not achieved (7, 13).

**TABLE 3:** Breakpoints used for drug susceptibility of testing rapidly growing mycobacteria by broth dilution^*a*^

Antibiotic	MIC (μg/mL)			Broth dilution range (μg/mL)
	Susceptible	Intermediate	Resistant	
Clarithromycin	≤2	4	≥8	0.06–64
Azithromycin	≤2	4	≥8	0.06**–**64
Amikacin	≤16	32	≥64	4**–**128
Tobramycin	≤2	4	≥8	0.5**–**16
Imipenem	≤4	8–16	≥32	2**–**64
Meropenem	≤4	8–16	≥32	2**–**64
Faropenem	N/A	N/A	N/A	2**–**64
Levofloxacin	≤1	2	≥4	1**–**32
Moxifloxacin	≤1	2	≥4	0.25**–**8
Sitafloxacin	N/A	N/A	N/A	0.25**–**8
Trimethoprim/sulfamethoxazole	≤38/2	N/A	≥76/4	4.8/0.25**–**152/8
Doxycycline	≤1	2–4	≥8	0.5**–**16
Linezolid	≤8	16	≥32	1**–**32
Clofazimine	N/A	N/A	N/A	0.06**–**2

a MIC, minimum inhibitory concentration; N/A, not applicable.

### Patient demographics.

Patient demographic data, including sex, age, body mass index, smoking history, comorbidities, radiographic findings, and laboratory findings, were collected. Radiological findings were categorized into four types based on chest computed tomography: fibrocavitary type, cavitary nodular bronchiectasis (NB) type, noncavitary NB type, and unclassified type (36).

### Statistical analysis.

All statistical analyses were conducted using R version 4.0.3 (R Foundation for Statistical Computing, Vienna, Austria). All data are shown as medians (interquartile ranges) for continuous variables and numbers (percentages) for categorical variables. Differences in data between groups were compared using the Mann-Whitney U test (continuous variables) or Fisher's exact test (categorical variables). A *P* value <0.05 was defined as significant.

## References

[1] Prevots DR, Marras TK. 2015. Epidemiology of human pulmonary infection with nontuberculous mycobacteria: a review. Clin Chest Med 36:13–34. doi:10.1016/j.ccm.2014.10.002. 25676516 PMC4332564

[2] Morimoto K, Hasegawa N, Izumi K, Namkoong H, Uchimura K, Yoshiyama T, Hoshino Y, Kurashima A, Sokunaga J, Shibuya S, Shimojima M, Ato M, Mitarai S. 2017. A laboratory-based analysis of nontuberculous mycobacterial lung disease in Japan from 2012 to 2013. Ann Am Thorac Soc 14:49–56. doi:10.1513/AnnalsATS.201607-573OC. 27788025

[3] Kwak N, Dalcolmo MP, Daley CL, Eather G, Gayoso R, Hasegawa N, Jhun BW, Koh WJ, Namkoong H, Park J, Thomson R, van Ingen J, Zweijpfenning SMH, Yim JJ. 2019. *Mycobacterium abscessus* pulmonary disease: individual patient data meta-analysis. Eur Respir J 54:1801991. doi:10.1183/13993003.01991-2018. 30880280

[4] Pasipanodya JG, Ogbonna D, Ferro BE, Magombedze G, Srivastava S, Deshpande D, Gumbo T. 2017. Systematic review and meta-analyses of the effect of chemotherapy on pulmonary *Mycobacterium abscessus* outcomes and disease recurrence. Antimicrob Agents Chemother 61:e01206-17. doi:10.1128/AAC.01206-17. 28807911 PMC5655093

[5] Koh WJ, Jeon K, Lee NY, Kim BJ, Kook YH, Lee SH, Park YK, Kim CK, Shin SJ, Huitt GA, Daley CL, Kwon OJ. 2011. Clinical significance of differentiation of *Mycobacterium massiliense* from *Mycobacterium abscessus*. Am J Respir Crit Care Med 183:405–410. doi:10.1164/rccm.201003-0395OC. 20833823

[6] Koh WJ, Jeong BH, Kim SY, Jeon K, Park KU, Jhun BW, Lee H, Park HY, Kim DH, Huh HJ, Ki CS, Lee NY, Kim HK, Choi YS, Kim J, Lee SH, Kim CK, Shin SJ, Daley CL, Kim H, Kwon OJ. 2017. *Mycobacterial characteristics* and treatment outcomes in *Mycobacterium abscessus* lung disease. Clin Infect Dis 64:309–316. doi:10.1093/cid/ciw724. 28011608

[7] Daley CL, Iaccarino JM, Lange C, Cambau E, Wallace RJ, Andrejak C, Böttger EC, Brozek J, Griffith DE, Guglielmetti L, Huitt GA, Knight SL, Leitman P, Marras TK, Olivier KN, Santin M, Stout JE, Tortoli E, van Ingen J, Wagner D, Winthrop KL. 2020. Treatment of nontuberculous mycobacterial pulmonary disease: An Official ATS/ERS/ESCMID/IDSA Clinical Practice Guideline. Clin Infect Dis 71:905–913. doi:10.1093/cid/ciaa1125. 32797222 PMC7768745

[8] Nessar R, Cambau E, Reyrat JM, Murray A, Gicquel B. 2012. *Mycobacterium abscessus*: a new antibiotic nightmare. J Antimicrob Chemother 67:810–818. doi:10.1093/jac/dkr578. 22290346

[9] Griffith DE, Aksamit T, Brown-Elliott BA, Catanzaro A, Daley C, Gordin F, Holland SM, Horsburgh R, Huitt G, Iademarco MF, Iseman M, Olivier K, Ruoss S, von Reyn CF, Wallace RJ, Jr, Winthrop K, Infectious Disease Society of America. 2007. An official ATS/IDSA statement: diagnosis, treatment, and prevention of nontuberculous mycobacterial diseases. Am J Respir Crit Care Med 175:367–416. doi:10.1164/rccm.200604-571ST. 17277290

[10] Brown-Elliott BA, Woods GL. 2019. Antimycobacterial susceptibility testing of nontuberculous mycobacteria. J Clin Microbiol 57:e00834-19. doi:10.1128/JCM.00834-19. 31315954 PMC6760954

[11] Aono A, Morimoto K, Chikamatsu K, Yamada H, Igarashi Y, Murase Y, Takaki A, Mitarai S. 2019. Antimicrobial susceptibility testing of *Mycobacteroides (Mycobacterium) abscessus* complex, *Mycolicibacterium (Mycobacterium) fortuitum*, and *Mycobacteroides (Mycobacterium) chelonae*. J Infect Chemother 25:117–123. doi:10.1016/j.jiac.2018.10.010. 30447882

[12] Kamada K, Yoshida A, Iguchi S, Arai Y, Uzawa Y, Konno S, Shimojima M, Kikuchi K. 2021. Nationwide surveillance of antimicrobial susceptibility of 509 rapidly growing mycobacteria strains isolated from clinical specimens in Japan. Sci Rep 11:12208. doi:10.1038/s41598-021-91757-4. 34108590 PMC8190260

[13] van Ingen J, Aksamit T, Andrejak C, Böttger EC, Cambau E, Daley CL, Griffith DE, Guglielmetti L, Holland SM, Huitt GA, Koh WJ, Lange C, Leitman P, Marras TK, Morimoto K, Olivier KN, Santin M, Stout JE, Thomson R, Tortoli E, Wallace RJ, Jr, Winthrop KL, Wagner D. 2018. Treatment outcome definitions in nontuberculous mycobacterial pulmonary disease: an NTM-NET consensus statement. Eur Respir J 51:1800170. doi:10.1183/13993003.00170-2018. 29567726 PMC6660914

[14] Maurer FP, Castelberg C, Quiblier C, Böttger EC, Somoskövi A. 2014. Erm(41)-dependent inducible resistance to azithromycin and clarithromycin in clinical isolates of *Mycobacterium abscessus*. J Antimicrob Chemother 69:1559–1563. doi:10.1093/jac/dku007. 24500188

[15] Bernut A, Le Moigne V, Lesne T, Lutfalla G, Herrmann JL, Kremer L. 2014. In vivo assessment of drug efficacy against *Mycobacterium abscessus* using the embryonic zebrafish test system. Antimicrob Agents Chemother 58:4054–4063. doi:10.1128/AAC.00142-14. 24798271 PMC4068527

[16] Shirata M, Yoshimoto Y, Marumo S, Tamai K, Fukui M. 2020. In vitro efficacy of combinations of eight antimicrobial agents against *Mycobacteroides abscessus* complex. Int J Infect Dis 97:270–277. doi:10.1016/j.ijid.2020.06.007. 32526389

[17] Haworth CS, Banks J, Capstick T, Fisher AJ, Gorsuch T, Laurenson IF, Leitch A, Loebinger MR, Milburn HJ, Nightingale M, Ormerod P, Shingadia D, Smith D, Whitehead N, Wilson R, Floto RA. 2017. British Thoracic Society guidelines for the management of non-tuberculous mycobacterial pulmonary disease (NTM-PD). Thorax 72:ii1–ii64. doi:10.1136/thoraxjnl-2017-210927. 29054853

[18] Chen J, Zhao L, Mao Y, Ye M, Guo Q, Zhang Y, Xu L, Zhang Z, Li B, Chu H. 2019. Clinical efficacy and adverse effects of antibiotics used to treat *Mycobacterium abscessus* pulmonary disease. Front Microbiol 10:1977. doi:10.3389/fmicb.2019.01977. 31507579 PMC6716072

[19] Yi L, Yoshiyama T, Okumura M, Morimoto K, Sasaki Y, Shiraishi Y, Ogata H, Mitarai S. 2017. Linezolid as a potentially effective drug for the treatment of multidrug-resistant tuberculosis in Japan. Jpn J Infect Dis 70:96–99. doi:10.7883/yoken.JJID.2015.629. 27000461

[20] Winthrop KL, Ku JH, Marras TK, Griffith DE, Daley CL, Olivier KN, Aksamit TR, Varley CD, Mackey K, Prevots DR. 2015. The tolerability of linezolid in the treatment of nontuberculous mycobacterial disease. Eur Respir J 45:1177–1179. doi:10.1183/09031936.00169114. 25614169 PMC6660918

[21] Tomioka H, Sato K, Akaki T, Kajitani H, Kawahara S, Sakatani M. 1999. Comparative in vitro antimicrobial activities of the newly synthesized quinolone HSR-903, sitafloxacin (DU-6859a), gatifloxacin (AM-1155), and levofloxacin against *Mycobacterium tuberculosis* and *Mycobacterium avium* complex. Antimicrob Agents Chemother 43:3001–3004. doi:10.1128/AAC.43.12.3001. 10582897 PMC89602

[22] Sano C, Tatano Y, Shimizu T, Yamabe S, Sato K, Tomioka H. 2011. Comparative in vitro and in vivo antimicrobial activities of sitafloxacin, gatifloxacin and moxifloxacin against *Mycobacterium avium*. Int J Antimicrob Agents 37:296–301. doi:10.1016/j.ijantimicag.2010.12.014. 21353489

[23] Asakura T, Suzuki S, Fukano H, Okamori S, Kusumoto T, Uwamino Y, Ogawa T, So M, Uno S, Namkoong H, Yoshida M, Kamata H, Ishii M, Nishimura T, Hoshino Y, Hasegawa N. 2019. Sitafloxacin-containing regimen for the treatment of refractory *Mycobacterium avium* complex lung disease. Open Forum Infect Dis 6:ofz108. doi:10.1093/ofid/ofz108. 31111076 PMC6519390

[24] Yang B, Jhun BW, Moon SM, Lee H, Park HY, Jeon K, Kim DH, Kim SY, Shin SJ, Daley CL, Koh WJ. 2017. Clofazimine-containing regimen for the treatment of *Mycobacterium abscessus* lung disease. Antimicrob Agents Chemother 61:e02052-16. doi:10.1128/AAC.02052-16. 28348153 PMC5444135

[25] Kwak N, Whang J, Yang JS, Kim TS, Kim SA, Yim JJ. 2021. Minimal inhibitory concentration of clofazimine among clinical isolates of nontuberculous mycobacteria and its impact on treatment outcome. Chest 159:517–523. doi:10.1016/j.chest.2020.07.040. 32712225

[26] Ferro BE, Meletiadis J, Wattenberg M, de Jong A, van Soolingen D, Mouton JW, van Ingen J. 2016. Clofazimine prevents the regrowth of *Mycobacterium abscessus* and *Mycobacterium avium* type strains exposed to amikacin and clarithromycin. Antimicrob Agents Chemother 60:1097–1105. doi:10.1128/AAC.02615-15. 26643335 PMC4750661

[27] Martiniano SL, Wagner BD, Levin A, Nick JA, Sagel SD, Daley CL. 2017. Safety and effectiveness of clofazimine for primary and refractory nontuberculous mycobacterial infection. Chest 152:800–809. doi:10.1016/j.chest.2017.04.175. 28483608

[28] Tanaka E, Kimoto T, Tsuyuguchi K, Suzuki K, Amitani R. 2002. Successful treatment with faropenem and clarithromycin of pulmonary Mycobacterium abscessus infection. J Infect Chemother 8:252–255. doi:10.1007/s10156-002-0176-8. 12373490

[29] Story-Roller E, Maggioncalda EC, Lamichhane G. 2019. Select β-lactam combinations exhibit synergy against *Mycobacterium abscessus* in vitro. Antimicrob Agents Chemother 63:e02613-18. doi:10.1128/AAC.02613-18. 30745389 PMC6437493

[30] Jhun BW, Kim SY, Moon SM, Jeon K, Kwon OJ, Huh HJ, Ki CS, Lee NY, Shin SJ, Daley CL, Koh WJ. 2018. Development of macrolide resistance and reinfection in refractory mycobacterium avium complex lung disease. Am J Respir Crit Care Med 198:1322–1330. doi:10.1164/rccm.201802-0321OC. 29877739

[31] Rominski A, Schulthess B, Müller DM, Keller PM, Sander P. 2017. Effect of β-lactamase production and β-lactam instability on MIC testing results for *Mycobacterium abscessus*. J Antimicrob Chemother 72:3070–3078. doi:10.1093/jac/dkx284. 28961987

[32] Morimoto K, Nakagawa T, Asami T, Morino E, Fujiwara H, Hase I, Tsujimoto Y, Izumi K, Hayashi Y, Matsuda S, Murase Y, Yano R, Takasaki J, Betsuyaku T, Aono A, Goto H, Nishimura T, Sasaki Y, Hoshino Y, Kurashima A, Ato M, Ogawa K, Hasegawa N, Mitarai S. 2018. Clinico-microbiological analysis of 121 patients with pulmonary *Mycobacterium abscessus* complex disease in Japan - An NTM-JRC study with RIT. Respir Med 145:14–20. doi:10.1016/j.rmed.2018.10.012. 30509703

[33] Patel R. 2015. MALDI-TOF MS for the diagnosis of infectious diseases. Clin Chem 61:100–111. doi:10.1373/clinchem.2014.221770. 25278500

[34] Nakanaga K, Sekizuka T, Fukano H, Sakakibara Y, Takeuchi F, Wada S, Ishii N, Makino M, Kuroda M, Hoshino Y. 2014. Discrimination of *Mycobacterium abscessus* subsp. *massiliense* from *Mycobacterium abscessus* subsp. *abscessus* in clinical isolates by multiplex PCR. J Clin Microbiol 52:251–259. doi:10.1128/JCM.01327-13. 24197885 PMC3911466

[35] Pang H, Li G, Zhao X, Liu H, Wan K, Yu P. 2015. Drug susceptibility testing of 31 antimicrobial agents on rapidly growing mycobacteria Isolates from China. Biomed Res Int 2015:419392. doi:10.1155/2015/419392. 26351633 PMC4550772

[36] Furuuchi K, Fujiwara K, Uesgi F, Shimoda M, Seto S, Tanaka Y, Yoshiyama T, Yoshimori K, Kurashima A, Ohta K, Morimoto K. 2021. Posttreatment lymphopenia is associated with an increased risk of redeveloping nontuberculous lung disease in patients with *Mycobacterium avium* complex lung disease. Clin Infect Dis 73:e152–e157. doi:10.1093/cid/ciaa729. 32507892

