# Supplementary material for: Minimum Inhibitory Concentrations before and after Antibacterial Treatment in Patients with Mycobacterium abscessus Pulmonary Disease
Source: Microbiol Spectr. 2021 Dec 8;9(3):e01928-21. doi: 10.1128/Spectrum.01928-21 (PMC8653840; doi:10.1128/Spectrum.01928-21)
Supplement: SUPPLEMENTAL FILE 1 — Supplemental material. Download SPECTRUM01928-21_Supp_1_seq7.pdf, PDF file, 0.1 MB [file spectrum01928-21_supp_1_seq7.pdf]

Table S1. Baseline characteristics of RGM patients other than MABS

Characteristic	*M. fortuitum* (n = 3)	*M. chelonae* (n = 1)	*M. mageritense* (n = 1)	*M. mucogenicum* (n = 1)
Female	3 (100)	1 (100)	0 (0.0)	0 (0.0)
Age, median	65 (62–66)	71	82	77
Body mass index, kg/m2	20.1 (20.1–20.4)	15.3	21.3	13.2
Smoking history				
Never smoker	2 (66.7)	1 (100)	0 (0.0)	1 (100)
Respiratory disease				
Sequel of tuberculosis	0 (0.0)	0 (0.0)	1 (100)	0 (0.0)
Previous NTM pulmonary disease	2 (66.7)	0 (0.0)	0 (0.0)	1 (100)
Aspergillus	0 (0.0)	1 (100)	0 (0.0)	0 (0.0)
Systemic disease				
Diabetes Mellitus	0 (0.0)	0 (0.0)	1 (100)	0 (0.0)
Gastro-intestinal disease	1 (33.3)	0 (0.0)	0 (0.0)	0 (0.0)
Radiographic findings				
Classification				
Non-cavitary NB	2 (66.7)	0 (0.0)	0 (0.0)	0 (0.0)
Cavitary NB	1 (33.3)	0 (0.0)	0 (0.0)	1 (100)
Fibrocavitary	0 (0.0)	1 (100)	0 (0.0)	0 (0.0)
Unclassified	0 (0.0)	0 (0.0)	1 (100)	0 (0.0)
Positive AFB smear	3 (100)	0 (0.0)	1 (100)	0 (0.0)

Data are presented as n (%) or median (interquartile). RGM = rapidly growing mycobacteria, MABS = *Mycobacterium abscessus*, *M. fortuitum* = *Mycobacterium fortuitum*, *M. chelonae* = *Mycobacterium chelonae*, *M. mageritense* = *Mycobacterium mageritense*, *M. mucogenicum* = *Mycobacterium mucogenicum*, NTM = nontuberculous mycobacteria, NB = Nodular bronchiectatic, AFB = Acid-fast bacilli.
